# Hemopoietic stem cell transplantation for infectious mononucleosis-related aplastic anemia

**DOI:** 10.1007/s00277-026-06746-2

**Published:** 2026-03-04

**Authors:** Stefano Cordella, Andrea Gilioli, Valeria Pioli, Andrea Messerotti, Fabio Forghieri, Francesca Bettelli, Monica Morselli, Luca Cassanelli, Leonardo Potenza, Roberto Marasca, Paola Bresciani, Angela Cuoghi, Mario Luppi

**Affiliations:** 1 Clinical and Experimental Medicine PhD Programme, Department of Biomedical, Metabolic and Neural Sciences, University of Modena and Reggio Emilia, Modena, 41125 Italy; 2 Hematology Unit, Azienda Ospedaliero-Universitaria, Modena, 41124 Italy; 3 Department of Medical and Surgical Sciences, Section of Hematology, University of Modena and Reggio Emilia, Modena, 41124 Italy

**Keywords:** Aplastic anemia, Epstein–Barr virus, Bone marrow failure, Pancytopenia, Allogeneic stem cell transplantation, EBV reactivation

## Abstract

We report the case of a young woman who developed aplastic anemia (AA), following a serologically confirmed primary Epstein–Barr virus (EBV) infection, occurring with fever and pharyngotonsillitis, in the absence of either palpable lymph nodes or enlarged spleen. Pancytopenia persisted after EBV DNA clearance, requiring multiple red blood cell and platelet transfusions. Given the availability of a human leukocyte antigen (HLA)-matched sibling donor (MSD), hematopoietic stem cell transplantation (HSCT) from bone marrow source was performed after a non-myeloablative conditioning regimen with cyclophosphamide and thymoglobulin. Graft-versus-host disease (GVHD) prophylaxis consisted of cyclosporine A and methotrexate. EBV reactivation occurred, one month post-HSCT, peaking at 28,838 DNA copies/ml in peripheral blood, without evidence of post-transplant lymphoproliferative disorder. Two pre-emptive doses of rituximab were administered, resulting in sustained EBV DNA negativity. Subsequent bone marrow evaluation showed normal cellularity and restoration of peripheral counts. After two years of follow-up, the patient remains transfusion-independent, with stable hematologic recovery, no signs of GVHD, and persistent mixed chimerism (70–75% host cells in peripheral blood; about 60% donor CD3 + lymphocytes). To our knowledge, this is the only second reported case of EBV-related AA successfully treated with MSD HSCT. This case underscores the importance of assessing EBV serology in all patients with AA, since EBV infection may be mild or subclinical, and highlights the efficacy of early rituximab administration in high-level EBV DNA reactivation after transplantation.

## Introduction

Bone marrow failure syndromes have been proposed to be pathogenetically associated with viral infections [1]. However, convincing descriptions of a clear-cut viral etiology of bone marrow aplasia in immunocompetent individuals are very rare and treatment has ranged from immunosuppressive therapy to hematopoietic stem cell transplantation (HSCT), with variable responses.

Moreover, the differential diagnosis is often challenging due to the nonspecific presentation, typically characterized by fever and cytopenias, which may overlap with manifestations ofhyperinflammatory conditions such as hemophagocytic lymphohistiocytosis (HLH) [2].

## Case presentation

**Fig. 1:**
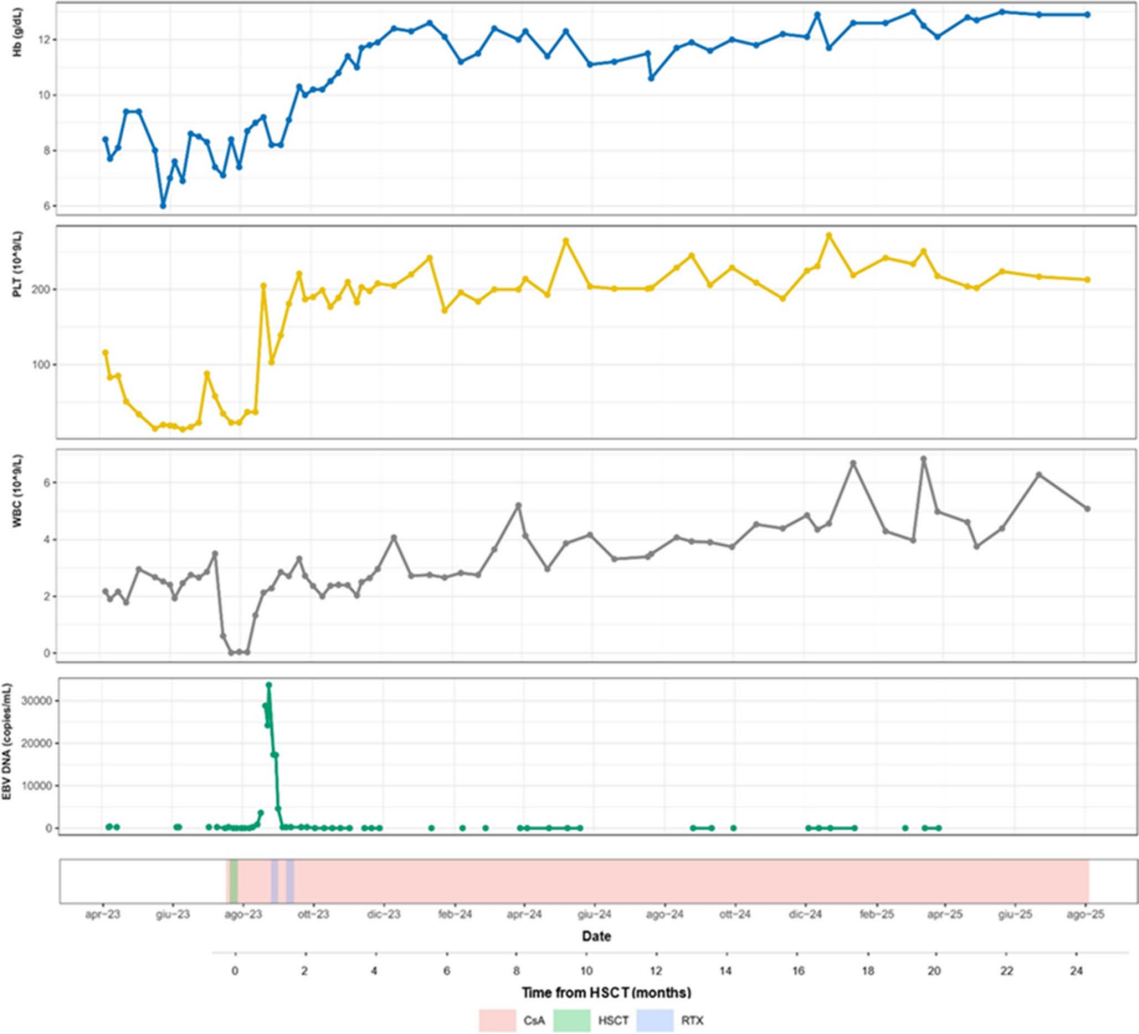
Changes in peripheral blood counts and different treatments. CsA, cyclosporine A; HSCT, hematopoietic stem cell transplantation; RTX, rituximab

We report the case of a 22-year-old Caucasian woman admitted to our Hematology Division in April 2023 for severe pancytopenia, associated with fever and pharyngotonsillitis, begun about one month earlier, in the absence of either palpable lymph nodes or enlarged spleen on physical examination by the general practitioner. Previous medical history was insignificant. Upon admission, complete blood count (CBC) revealed a white blood cell (WBC) count of 2.17 × 10⁹/L without circulating blasts, neutrophils 0.29 × 10⁹/L, hemoglobin (Hb) 8.4 g/dl with mean corpuscular volume (MCV) 88.9 fl., reticulocytes 24 × 10⁹/L and platelet (Plt) count 116 × 10⁹/L (Figure 1). No signs of coagulopathy were present, and C-reactive protein (CRP) was 6.9 mg/dl. Physical examination was unremarkable. Liver function tests were normal. Vitamin B12 and folate levels were in normal ranges. Imaging studies, including abdominal ultrasound and computed tomography scan of the neck-chest-abdomen, showed neither hepatosplenomegaly nor lymphadenopathies, nor other abnormalities. Diepoxy butane (DEB) test was negative. To investigate the etiology of pancytopenia, morphological, cytochemical, molecular and immunophenotypic analyses on peripheral blood (PB) and bone marrow aspirate were performed. They demonstrated severe marrow hypoplasia (< 10% cellularity) without fibrosis, with neither blast excess, nor dysplasia. Immunophenotyping showed a relative increase of T-lymphocytes (CD38+, DR−, CD45R0−/+, with an equal distribution between CD4 + and CD8 + subsets), and no paroxysmal nocturnal hemoglobinuria clone was detected. Conventional cytogenetics reported a normal female karyotype (46, XX). Polymerase chain reaction (PCR) and neologyxt generation sequencing assays did not identify either mutations or fusion transcripts associated with myeloid neoplasms.

HLH was excluded based on bone marrow morphologic examination, and the absence of both clinical and radiological signs of hepatosplenomegaly, the normal ferritin and triglyceride levels, and the absence of hypofibrinogenemia (fibrinogen consistently > 150 mg/dL). The HS score was 67 not consistent with a diagnosis of HLH [3, 4].

Moreover, autoimmune screening tests (antinuclear antibodies, rheumatoid factor, antineutrophil cytoplasmic antibodies, Coombs test) were negative. Viral and microbiological assays were performed and resulted negative for cytomegalovirus, human immunodeficiency virus, hepatitis A, B, C viruses, parvovirus B19, and Leishmania, whereas Epstein-Barr virus (EBV) serology showed positive Viral Capsid Antigen (VCA) IgM ang IgG antibodies, with negative Epstein Barr Nuclear Antigen (EBNA) antibodies, and PCR for EBV DNA was positive both in the bone marrow (397 copies/ml) and the peripheral blood (225 copies/ml), consistent with recent primary EBV infection.

In July 2023, EBV seroconversion occurred and EBV DNA was undetectable on peripheral blood. However, pancytopenia persisted (WBC 3.88 × 10⁹/L, N 1.7 × 10⁹/L, Hb 8 g/dl, Plt 28 × 10⁹/L, reticulocytes 29 × 10⁹/L), and the patient required multiple red blood cell and platelet transfusions. A diagnosis of severe aplastic anemia (SAA) following recent primary EBV infection was made. Given the availability of an HLA-matched sibling donor (MSD), HSCT was performed, using bone marrow (BM) as stem cell source (total cell dose 2.37 × 10e6/kg CD34+), after non-myeloablative conditioning regimen with cyclophosphamide total 200 mg/kg and rabbit ATG (Thymoglobulin) total 7 mg/kg (after an infusion reaction, preventing completion of the planned 7.5 mg/kg dose). Cyclosporine A (CsA) and methotrexate (10 mg/m2 days 1, 3, 6, after HSCT) were adopted as Graft-versus-Host Disease (GVHD) prophylaxis. Neutrophil engraftment (> 500/ul) was reached at day + 24 after transplant, and Plt engraftment (> 20.000/ul) was reached at day + 18. EBV DNA was monitored on PB biweekly, in the first month after transplant, and monthly, for the following two months. At day + 24 post-HSCT, EBV reactivation was observed, peaking at day + 31 (DNA 28,838 copies/ml in peripheral blood) (Figure 1). No signs of post-transplant lymphoproliferative disorder (PTLD) were detected. Two pre-emptive doses of rituximab (375 mg/m²) were administered on days + 35 and + 42, resulting in stable EBV DNA negativity, thereafter. At day + 62 post-transplant, bone marrow evaluation showed normal cellularity and normalization of peripheral counts. Chimerism analysis revealed 34% donor cells in bone marrow and 12% donor CD3 + lymphocytes. CsA was maintained at therapeutic trough levels (> 250 ng/ml) for one year after transplant, then slowly tapered, and it continues to be administered, at last follow-up.

After 2-year of follow-up, the patient remains transfusion-independent, with stable hematologic recovery and no signs of graft-versus-host disease (GVHD). Chimerism analysis showed persistent mixed chimerism (70–75% host cells in peripheral blood; about 60% donor CD3 + lymphocytes) (Table 1) .

## Discussion

Bone marrow aplasia/hypoplasia following EBV-induced infectious mononucleosis is a recognized complication in X-linked lymphoproliferative syndrome [5, 6].

Furthermore, bone marrow failure and pancytopenia following primary EBV infection in immunocompetent individuals is an uncommon but clinically significant complication, with only few cases described in the literature. Baranski et al. described six patients with AA in whom EBV DNA and proteins were detected in the bone marrow. Notably, these patients exhibited a distinct pattern of EBV DNA fragment expression compared with that typically observed in infectious mononucleosis. All were treated with immunosuppressive therapy (IST)—anti-thymocyte globulin (ATG) alone or, in two cases, combined with CsA—together with acyclovir; no one underwent HSCT. Despite these interventions, three patients died from complications of pancytopenia, and the remaining three had persistent cytopenia of varying severity [7]. EBV-associated AA cases have been reported to show variable responses to IST. Grishaber et al., reported and revised the clinical course of a group of children with AA, following EBV infection, responsive to high dose prednisone [8]. Shadduck et al., reported a 17-year-old female who developed AA after infectious mononucleosis, successfully treated with ATG [9]. Similarly, Lazarus et al. described a 12-year-old girl who developed severe AA, three weeks after symptoms of infectious mononucleosis. In their literature review, the mean time from onset of infectious mononucleosis symptoms to the nadir of pancytopenia was 21.3 days (range, 7–49 days), and, in responders, the mean time from pancytopenia diagnosis to hematologic recovery was 6.25 days (range, 4–8 days), apparently confirming responsiveness to steroids in some cases [10]. Gunchenko et al. also documented a patient with EBV-associated hepatitis who subsequently developed AA, pernicious anemia, and autoimmune thyroiditis, showing hematologic improvement under IST [11].

Anderlini et al. reported the case of a 17-year-old girl with severe AA following infectious mononucleosis, who underwent a syngeneic blood stem cell transplantation with a “large stem cell dose” of freshly infused total of 10.2 × 106 CD34 + cells/kg without prior immunosuppression, achieving a transient trilineage hematological response, lasting only six months. Only after she received a second syngeneic stem cell infusion, after conditioning with cyclophosphamide and ATG, the patient achieved a durable hematological recovery, lasting for almost 16 months [12].

Apart from these reports, no standardized treatment algorithm exists for EBV-related AA. Current guidelines for severe AA management do not provide EBV-specific recommendations, and treatment is typically chosen according to indications for idiopathic AA [13–15]. Similarly, the pathogenesis of EBV-related AA is unknown. By studying 15 patients with AA, unrelated to EBV primary infection, Ben Hamza et al. recently proposed that EBV infection may trigger the expansion of virus-specific T-cell clones that, through molecular mimicry, eliminate hematopoietic stem cells [16]. Whether these mechanisms of immune-mediated destruction of hematopoietic progenitors do occur also in AA shortly preceded by EBV primary infection remains to be investigated. Moreover, Zhang et al. [17] demonstrated a correlation between EBV DNA copy number and both granzyme B/perforin expression and interferon-γ secretion by CD8⁺ T cells, as well as reduced CD8⁺ T-cell viability, thereby linking acute EBV infection to the immunopathogenesis and progression of SAA.

## Conclusion

To our knowledge, this is the second reported case of EBV-related AA successfully treated with MSD HSCT. The clinical presentation is instructive, highlighting that AA may follow a primary EBV infection, even mild or subclinical, and underlining the need to assess EBV serology invariably in all cases of AA. Knowledge of a preceding EBV infection prompted a strict post-transplant monitoring of EBV DNA in PB and early intervention with pre-emptive rituximab, effectively preventing either EBV-related PTLD or graft failure. Two years after HSCT, neither relapse of pancytopenia nor evidence of GVHD have occurred, with a stable mixed donor chimerism (Table 1).

In conclusion, this case highlights the potential role of HSCT in young patients with EBV-related SAA, when a MSD is available. Further research is required to define the optimal treatment strategies, the timing of HSCT and the role of IST, in the rare EBV-related bone marrow failure syndromes, occurring in the general population.

**Table 1:** Chimerism analysis: percent donor cells in total peripheral blood (PB) and in CD3+ lymphocytes (CD3+)

Date	Time from HSCT(days)	Time from HSCT(months)	PB (%)	CD3+ (%)
18/08/2023	28	1	77	61
28/08/2023	38	1	65	44
12/09/2023	53	2	43	17
21/09/2023	62	2	35	12
18/10/2023	89	3	27	14
28/11/2023	130	4	24	37
27/12/2023	159	5	25	57
25/01/2024	188	6	32	68
23/02/2024	217	7	36	67
29/03/2024	252	8	19	55
09/05/2024	293	10	29	67
26/08/2024	402	13	25	67
13/11/2024	481	14	28	66
10/02/2025	570	15	26	63
22/05/2025	671	16	30	54
23/06/2025	703	17	25	58
04/08/2025	745	18	27	55

## Data Availability

No datasets were generated or analysed during the current study.
